# QTL analysis of internode elongation in response to gibberellin in deepwater rice

**DOI:** 10.1093/aobpla/plu028

**Published:** 2014-06-19

**Authors:** Keisuke Nagai, Yuma Kondo, Takuya Kitaoka, Tomonori Noda, Takeshi Kuroha, Rosalyn B. Angeles-Shim, Hideshi Yasui, Atsushi Yoshimura, Motoyuki Ashikari

**Affiliations:** 1 Bioscience and Biotechnology Center, Nagoya University, Furo-cho, Chikusa, Nagoya, Aichi 464-8601, Japan; 2 Plant Breeding Laboratory, Kyushu University, 6-10-1 Hakozaki-ku, Higashi, Fukuoka 812-8581, Japan

**Keywords:** Deepwater rice, gibberellin response, internode elongation, QTL.

## Abstract

Gibberellin (GA) is a plant hormone that has important roles in numerous plant developmental phases. Rice plants known as deepwater rice respond to flooding by elongating their internodes to avoid anoxia. Previous studies reported that GA is essential for internode elongation in deepwater rice. Quantitative trait locus (QTL) analyses identified QTLs regulating internode elongation in response to deepwater conditions. However, the interaction between internode elongation and regulators of GA sensitivity in deepwater rice is unknown. In this study, we applied GA to recombinant inbred lines of T65 (non-deepwater rice) and Bhadua (deepwater rice), and performed a QTL analysis of internode elongation in response to GA. GA-induced internode elongation was detected only in deepwater rice. Our QTL analysis revealed two major QTLs on chromosomes 3 and 9 regulating total internode length, lowest elongated internode and number of elongated internodes. Furthermore, the QTL on chromosome 3 acted as an enhancer of other QTLs (e.g. the QTL on chromosome 12). Nearly isogenic lines of deepwater rice carrying the QTL regions from chromosomes 3 and 12 of the deepwater rice C9285 showed internode elongation in response to GA. Thus, these QTLs may regulate GA responsiveness in deepwater rice. This study furthers our understanding of the mechanism of internode elongation in rice.

## Abstract (precis)

Gibberellin (GA) is one of the plant hormones which regulates many aspects of plant growth and developmental processes. Rice plants known as deepwater rice can survive during flooding by elongating its internodes to avoid anoxia. Previous studies reported that GA is essential for internode elongation in deepwater rice. However, the interaction between internode elongation and regulator of GA sensitivity is unknown. In this study, we performed a QTL analysis and identified the chromosomal regions that regulate GA responsiveness in deepwater rice. We concluded that deepwater rice could induce internode elongation in response to GA by factors in these regions.

## Introduction

The plant hormone gibberellin (GA) regulates many aspects of plant growth, organ development and environmental responses (Achard and Genschik 2009). Among its various functions, GA was initially identified to be involved in the phenomenon of stem and leaf elongation (Hori 1898; Sawada 1912; Kurosawa 1926). In this process of elongation, GA is associated with the regulation of cell division and cell elongation. In non-deepwater rice, GA biosynthesis and signalling occur in elongating and dividing organs and tissues, such as the shoot apex and young leaves (Kaneko *et al.* 2003). Additionally, rice internodes start to elongate after initiation of reproductive growth, and expression of genes involved in GA biosynthesis and signal transduction is upregulated in the basal region of the elongating internodes (Kaneko *et al.* 2003). In contrast, rice plants known as deepwater rice can induce internode elongation during vegetative growth. Deepwater rice can be cultivated under deepwater conditions. The leaves and internodes of the plants elongate to help keep the top leaves above the water surface and avoid anoxia under deepwater conditions (Vergara and Mazaredo 1979; Catling 1992). Previous physiological studies have suggested that GA is involved in deepwater rice elongation. GA treatment induces internode elongation in deepwater rice under normal conditions, and GA biosynthesis inhibitors (e.g. uniconazol and tetcyclacis) suppress internode elongation in deepwater rice (Raskin and Kende 1984; Inouye and Kim 1985; Hattori *et al.* 2009). Additionally, bioactive GA accumulates in deepwater rice under deepwater conditions, but not in non-deepwater rice (Hattori *et al.* 2009). These results suggest that GA is essential for internode elongation in deepwater rice. However, GA accumulation does not increase dramatically under deepwater conditions, and a rise in GA content is not sufficient to explain the marked internode elongation of deepwater rice. Deepwater rice may have not only greater GA biosynthesis ability but also higher GA sensitivity than non-deepwater rice. Previously, quantitative trait locus (QTL) analyses were performed to detect genetic loci that regulate the deepwater response. Multiple QTLs were detected on chromosomes 1, 3 and 12 by four independent groups using different populations (Nemoto *et al.* 2004; Tang *et al.* 2005; Hattori *et al.* 2007; Kawano *et al.* 2008). Moreover, we detected two QTLs on chromosomes 2 and 4 that enhance internode elongation during the early vegetative stage (Nagai *et al.* 2012). Among these QTLs, the QTL on chromosome 12, which is most effective for internode elongation, was found to contain *SNORKEL1* (*SK1*) and *SNORKEL2* (*SK2*) (Hattori *et al.* 2009). These genes encode ethylene-responsive factor-type transcription factors, and their expression is induced by ethylene. However, genes regulating internode elongation in deepwater rice in response to GA have not been identified. In the present study, we performed a QTL analysis of internode elongation in response to GA using recombinant inbred lines (RILs) of rice, Taichung 65 (T65; non-deepwater rice) and Bhadua (deepwater rice), and successfully detected QTLs. We also confirmed the effects of the detected QTLs using nearly isogenic lines (NILs) possessing the QTL regions of C9285, another deepwater rice, in the T65 genetic background.

## Methods

### Plant materials

T65 (*Oryza sativa* ssp. *japonica*), Nipponbare (*O. sativa* ssp. *japonica*), Bhadua (*O. sativa* ssp. *indica*; Bangladesh) and C9285 (*O. sativa* ssp. *indica*; Bangladesh) were used in this study. C9285 was kindly provided by the National Institute of Genetics in Japan (http://www.shigen.nig.ac.jp/rice/oryzabase/top/top.jsp) and maintained at Nagoya University. Nipponbare and T65 were maintained at Nagoya University. Recombinant inbred lines were produced by selfing (nine times) F_1_ plants derived from a cross between T65 and Bhadua. NIL3, NIL12 and NIL3–12 possessing the deepwater response QTL on chromosomes 3, 12 and 3/12 in the T65 genetic background were derived previously (Hattori *et al.* 2009).

### Plant growth conditions and GA treatment

Rice seeds were sterilized by boiling at 60 °C for 10 min. After sterilization, the seeds were allowed to germinate in water at 30 °C for 72 h, and then sown individually in a perforated cell tray (cell size, 2.5 × 2.5 × 4.5 cm) filled with soil. To eliminate the effect of endogenous GA, uniconazole (uni), an inhibitor of GA biosynthesis, was administered with or without GA. For GA and uni treatment, 2 mL of a 10^−1^ M GA_3_ stock solution and 0.2 mL of a 10^−2^ M uni stock solution were added to 2 L of water (final concentration, 10^−4^ M GA and 10^−6^ M uni). For uni treatment, 0.2 mL of a 10^−2^ M uni stock solution was added to 2 L of water (final concentration, 10^−6^ M uni). Each solution was used to treat 140 individuals. Seedlings were grown under 16 h of light and 8 h of dark, 70 % humidity, at 25 °C in a phytotron. After 3 weeks, the leaf sheath and internode elongation were measured.

### Phenotypic evaluation of total internode length, lowest elongated internode and number of elongated internodes

The developmental stage of a rice stem is defined as non-elongation phase, primary phase or secondary phase (Suetsugu 1968). Although internode elongation of <3 mm has been reported in primary phase stems, internode elongation exceeding 5 mm has been observed in secondary phase stems (Inouye *et al.* 1985). Deepwater rice enhances secondary phase growth, resulting in elongated internodes in response to deepwater conditions (Takahashi 1991). Based on these reports, we considered internodes longer than 5 mm to be elongated. The main culms of each plant were cut along the midline and total internode length (TIL) was measured. Because plants showing elongation of the lower internodes can increase TIL, lowest elongated internode (LEI) is regarded as an indicator of early internode elongation ability (Inouye *et al.* 1985; Ibi *et al.* 1995). Lowest elongated internode corresponds to the leaf number. In rice, the culm is characterized by structural superposition, created by the upper and lower nodes, a single internode and a single leaf (Arikado *et al.* 1990). Based on these reports, when internode elongation occurred from the internode of the third leaf in the main culm, LEI was considered to be 3. Number of elongated internodes (NEI) was defined as the number of internodes that are longer than 5 mm. The phenotype of each line was based on the average of six plants.

### Linkage map construction and QTL analysis

To construct a linkage map, genomic DNA was extracted from 84 individuals of the RIL using the isoplant method (Zhu *et al.* 1993). The extracted DNA samples were used for genotyping with single-nucleotide polymorphism (SNP) markers **[see Supporting Information]** using an Affymetrix customized SNP array for rice. Single-nucleotide polymorphisms were detected by the Golden Gate assay of BeadXpress (Illumina, Inc., San Diego, CA, USA). MAPMAKER/EXP, version 3.0 (Lander *et al.* 1987) was used to construct the linkage map. R/qtl (Broman and Sen 2009) was used for the QTL analysis. Composite interval mapping (CIM) was used to detect the QTLs. The number of covariates was assumed to be 1. Quantitative trait loci were considered significant at *P* < 0.05, with the logarithm of odds (LOD) score given by 1000 permutation tests (Churchill and Doerge 1994; Doerge and Churchill 1996). A two-QTL scan was performed for the detection of interacting QTLs. Quantitative trait loci were considered significant at *P* < 0.1 using the fitqtl method (Broman and Sen 2009). Candidate QTL regions were identified using the lodint method (Broman and Sen 2009).

## Results

### Response of T65, Nipponbare, Bhadua and C9285 to GA

**Figure 1.**
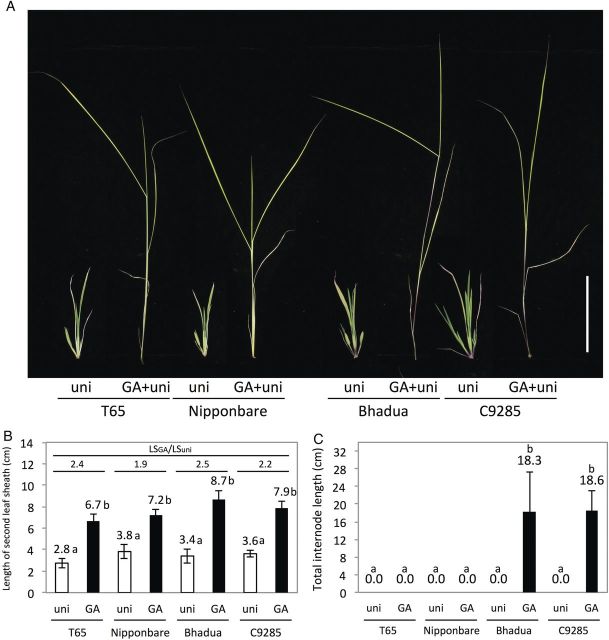
Phenotypic evaluation of GA-treated non-deepwater and deepwater rice. (A) Response of T65, Nipponbare, Bhadua and C9285 to GA and uni. (B) Length of the second leaf sheath in response to GA in non-deepwater rice and deepwater rice. LS_GA_/LS_uni_ is the ratio of the second leaf sheath length following GA treatment (LS_GA_) to the length with uni (LS_uni_). (C) Length of the internode in response to GA in non-deepwater rice and deepwater rice. Gibberellin and uni mean treatment with 10^−4^ M GA + 10^−6^ M uni and 10^−6^ M uni, respectively. Error bars indicate ± SD (*n* = 6). Different letters denote a significant difference (Tukey's test, *P* < 0.05).

Since GA is a key phytohormone in leaf and stem elongation, we compared the GA responsiveness of non-deepwater and deepwater rice. T65, Nipponbare, Bhadua and C9285 were used to confirm the elongation ability of rice in response to GA. After treatment with 10^−4^ M GA + 10^−6^ M uni or 10^−6^ M uni for 3 weeks, we measured the length of the second leaf sheaths and TIL (Fig. 1A–C). No clear difference was observed in the second leaf sheath (Fig. 1B). The ratios of the second Leaf Sheath length with GA treatment (LS_GA_) to the Leaf Sheath length with uni treatment (LS_uni_) (LS_GA_/LS_uni_) were 2.4, 1.9, 2.5 and 2.2, respectively. On the other hand, Bhadua and C9285 showed significant internode elongation in response to GA, but T65 and Nipponbare did not (Fig. 1C). These results suggest the presence of factors controlling internode elongation in response to GA in deepwater rice.

### Frequency distribution of TIL, LEI and NEI in T65/Bhadua RILs

**Figure 2.**
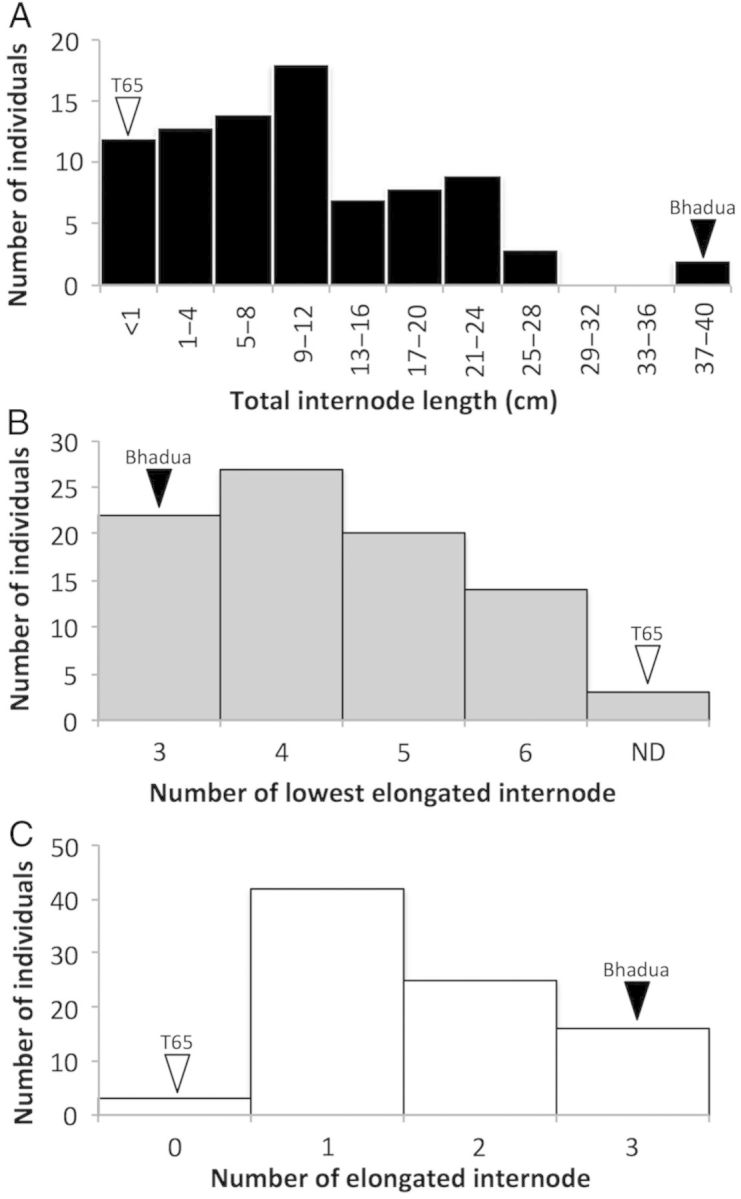
Distribution frequency of TIL, LEI and NEI among the RILs. After 3 weeks of GA treatment, TIL (A), LEI (B) and NEI (C) were measured. White and black arrowheads indicate the phenotypic values for T65 and Bhadua, respectively.

To identify QTLs controlling internode elongation under GA treatment, T65 and Bhadua RILs were used for QTL analysis. After GA treatment for 3 weeks, we measured TIL, LEI and NEI. The TIL and NEI values ranged from 0.0 to 40.0 cm and from 0 to 3, respectively (Fig. 2A and C). The LEI values ranged from 3 to 6; however, LEI in several lines could not be detected (ND) (Fig. 2B). These phenotypic values were distributed continuously between the average values of the parental plants, suggesting that there were no transgressive segregation events and that the phenotypes of the RIL are controlled by QTLs. We found a negative correlation between TIL and LEI (*P* < 0.01, *r* = −0.66) and between NEI and LEI (*P* < 0.01, *r* = −0.88). A positive correlation was found between TIL and NEI (*P* < 0.01, *r* = 0.74).

### QTL analysis of the T65/Bhadua RIL

**Figure 3.**
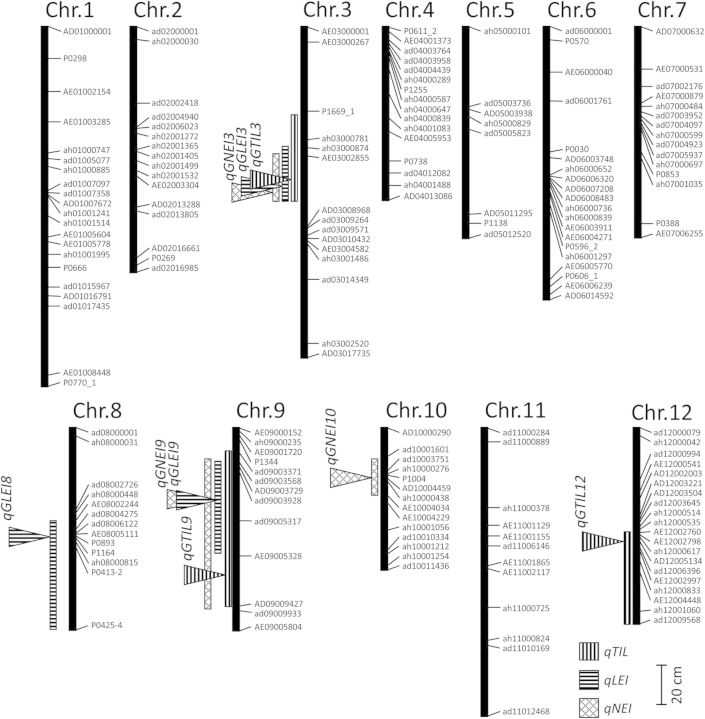
Linkage map for the RIL. Lines show the positions of the SNP markers used for the whole-genome survey. The vertical bars to the left of the maps indicate the candidate regions calculated by the lodint method in R/qtl. Arrowheads indicate the positions of the LOD peak for each QTL.

**Figure 4.**
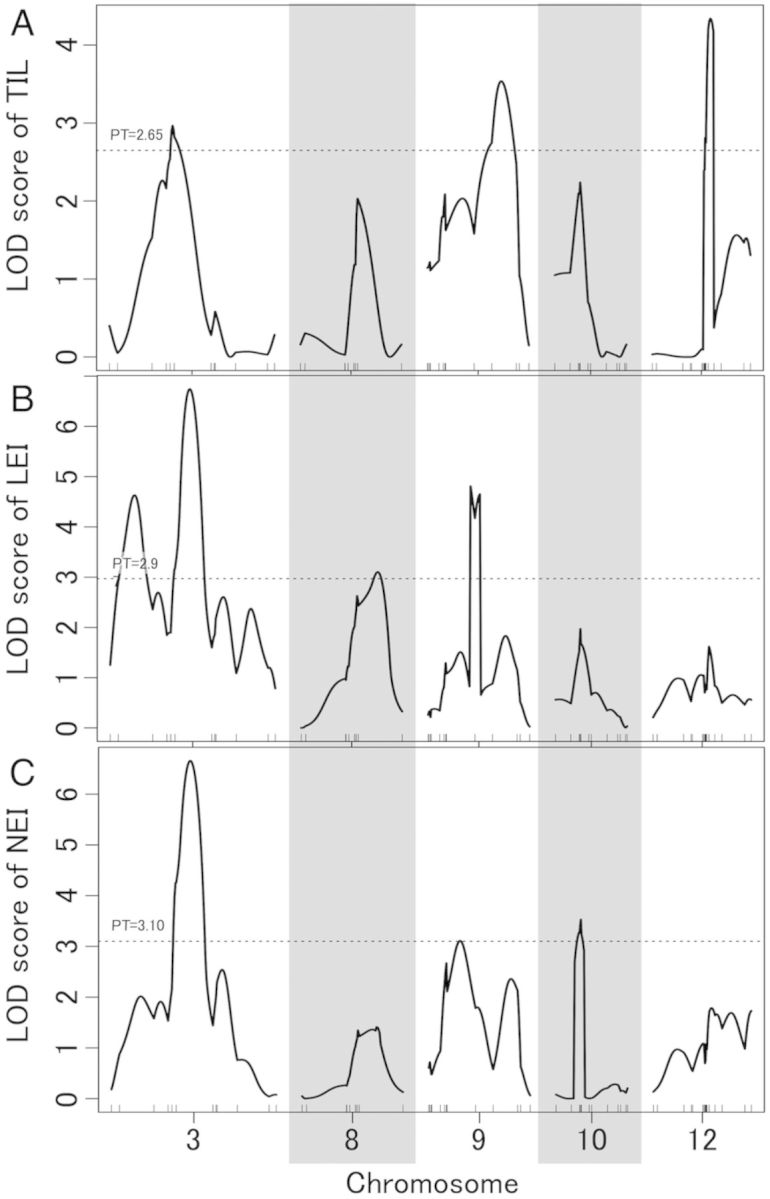
Quantitative trait loci for internode elongation in response to GA. (A) LOD score of TIL. (B) LOD score of LEI. (C) LOD score of NEI. Chromosomes with significant LOD values were selected. Dashed lines indicate the values of 1000 permutation tests (PT).

**Figure 5.**
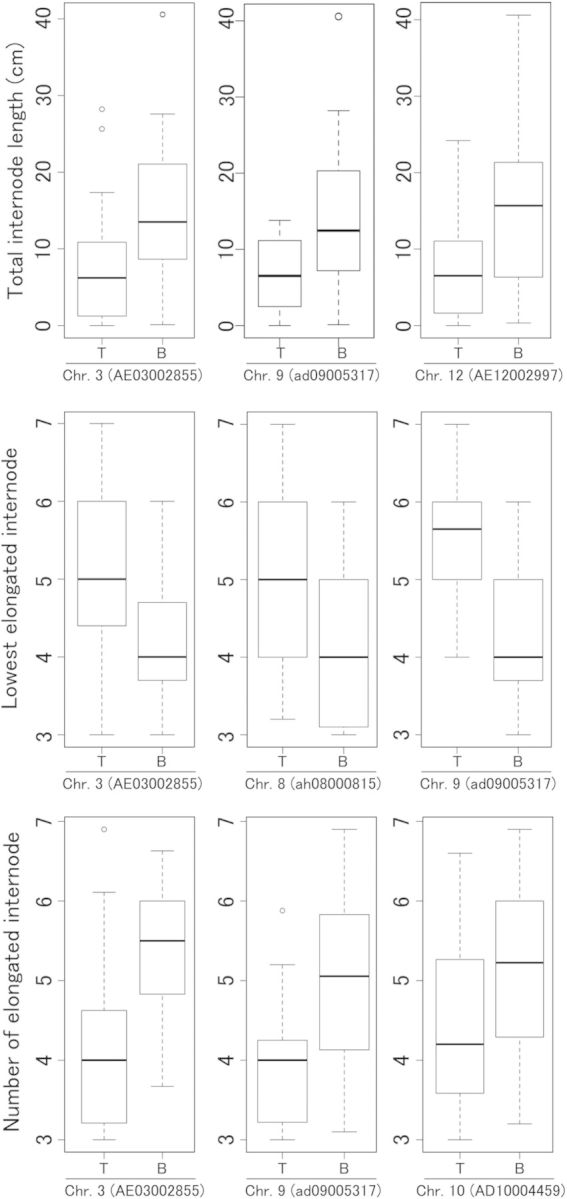
Box plots of the phenotypic values for each QTL. The box plots show the distribution of TIL, LEI and NEI value differences between T65 (T) and Bhadua (B), respectively. The values are derived from the phenotypic values at the SNP markers nearest to the peak QTLs.

**Figure 6.**
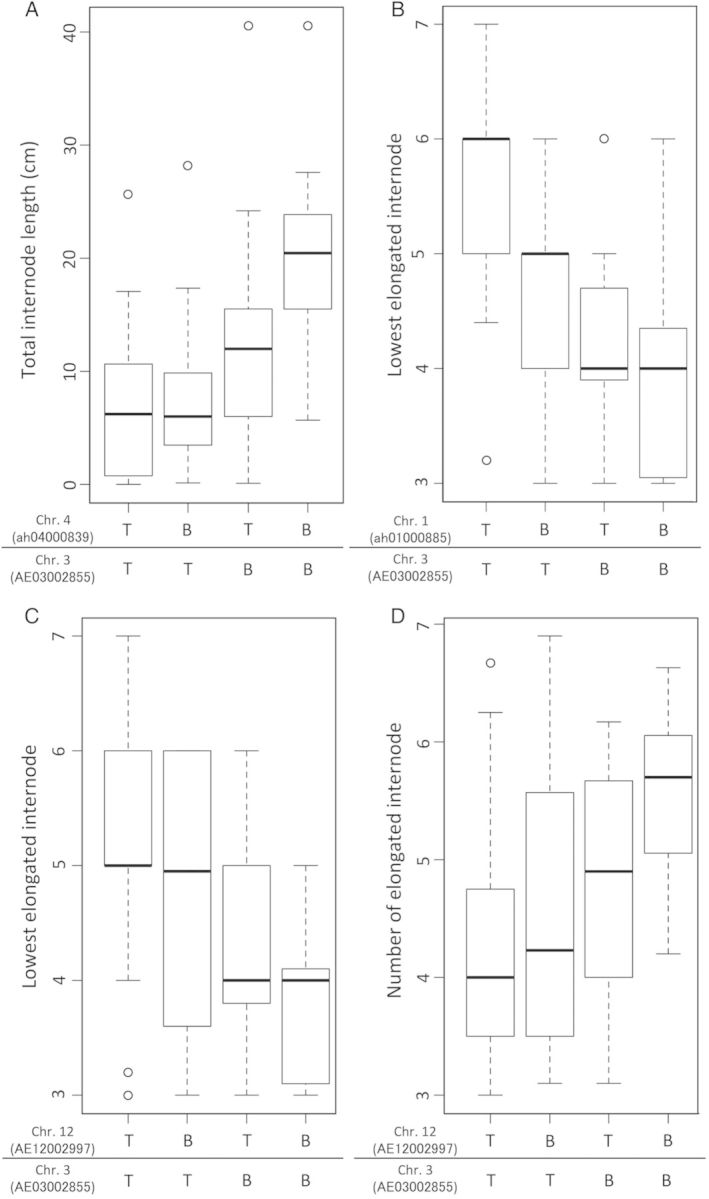
Box plots of the phenotypic values for QTL interactions. The box plots show the distribution of TIL, LEI and NEI value differences between combinations of the T65 (T) and Bhadua (B) genotypes. The values are derived from the phenotypic values at the SNP markers nearest to the peak QTLs.

A linkage map was constructed using the T65/Bhadua RIL. This map contained 183 SNP markers that covered the entire genome, with an average interval of 7.5 cM (Fig. 3). Using phenotypic values, as mentioned above, and our linkage map, we performed a QTL analysis for GA-responsive TIL, LEI and NEI (*qGTIL*, *qGLEI* and *qGNEI*). We determined the threshold values for LOD using 1000 permutations (2.65 for TIL, 2.97 for LEI, and 3.10 for NEI). Composite interval mapping for TIL detected significant peaks on chromosomes 3, 9 and 12 (*qGTIL3*, *qGTIL9* and *qGTIL12*), explaining 16.1, 9.6 and 20.8 % of the total phenotypic variance, respectively (Fig. 4A and Table 1). In an analysis of LEI, *qGLEI3* (LOD score, 6.74), *pGLEI8* (3.10) and *qLGEI9* (4.81) were identified on chromosomes 3, 8 and 9, respectively (Fig. 4B and Table 1). The total phenotypic variances for each QTL were 13.6, 14.5 and 16.7 %, respectively (Table 1). We also detected three QTLs for NEI on chromosomes 3, 9 and 10 (*qGNEI3*, LOD = 6.66; *qGNEI9*, 3.11; *qGNEI10*, 3.53), which explained 12.6, 10.8 and 17.9 % of the total phenotypic variance, respectively (Fig. 4C and Table 1). To evaluate the individual effects of each QTL, the phenotypic values of the RIL were separated on the basis of each QTL genotype (Fig. 5). Among the QTL regions, Bhadua homozygous class (B in Fig. 5) showed enhanced TIL, LEI and NEI phenotypic values, as compared with T65 homozygous class (T in Fig. 5), suggesting that these QTLs function as regulators of internode elongation in response to GA in Bhadua. Interestingly, the QTLs on chromosomes 3 and 9 were detected in similar regions across the three traits, implying the presence of major factors that control internode elongation in response to GA. To define other QTLs, we performed a two-QTL scan. Significant QTLs were found on chromosomes 1 (*qGTIL1*), 4 (*qGLEI4*) and 12 (*qGLEI12* and *qGNEI12*) at positions 70, 77 and 56 cM, respectively; all of these QTLs were detected together with the QTL on chromosome 3 (Table 2). Interactions between *qGTIL1* and *qGTIL3*, *qGLEI4* and *qGLEI3*, *qGLEI12* and *qGLEI3*, and *qGNEI12* and *qGNEI3* contributed 22.6, 20.6, 18.9 and 21.2 % to the phenotypic variance, respectively (Table 2). To evaluate the interactive effects of these combinations, the phenotypic values of the RIL were separated on the basis of each genotype in the QTL regions (Fig. 6). The individual effects of *qGTIL1*, *qGLEI4*, *qGLEI12* and *qGNEI12* were slight or not sufficient to enhance internode elongation, whereas the effects of the QTLs were promoted in combination with the QTL on chromosome 3 (Fig. 6). These results indicate that the QTL on chromosome 3 is required for the functioning of minor QTLs, and this QTL is a major factor controlling internode elongation in response to GA.

**Table 1.** Putative QTLs for TIL, LEI and NEI in response to GA. %var, rate of contribution for phenotype. Sig., significance of the QTL was determined by the fitqtl method in R/qtl at **P < 0.05.

Trait	QTL	Chr.	Position (cM)	Predicted region (cM)	Marker interval	LOD	%var	Sig.
TIL	*qGTIL3*	3	61	38–83	P1669_1–AD03008968	3.87	16.1	**
	*qGTIL9*	9	71	16–88	ad09003371–AD09009427	3.43	9.6	**
	*qGTIL12*	12	56	51–59	ah12000535–ah12001060	4.34	20.8	**
LEI	*qGLEI3*	3	77	68–87	AE03002855–AD03008968	6.74	13.6	**
	*qGLEI8*	8	74	47–85	AE08002244–P0425-4	3.10	14.5	**
	*qGLEI9*	9	41	40–51	ad09003928–AE09005328	4.81	16.7	**
NEI	*qGNEI3*	3	76	66–87	AE03002855–AD03008968	6.66	12.6	**
	*qGNEI9*	9	41	40–51	ad09003568–AD09009427	3.11	10.8	**
	*qGNEI10*	10	24	17–28	ad10001601–AE10004034	3.53	17.9	**

**Table 2.** Putative QTLs for TIL, LEI and NEI by the two-QTL scan. %var, rate of contribution for phenotype. Sig., significance of the QTL was determined by the fitqtl method in R/qtl at **P < 0.05 and *P < 0.1.

Trait	QTL	Chr.	Position (cM)	LOD	%var	Sig.
TIL	*qGTIL3*	3	70	4.68	22.6	**
	*qGTIL4*	4	35			
LEI	*qGLEI1*	1	77	4.22	20.6	*
	*qGLEI3*	3	75			
	*qGLEI3*	3	69	5.29	25.2	*
	*qGLEI12*	12	56			
NEI	*qGNEI3*	3	77	4.34	21.2	*
	*qGNEI12*	12	56

### QTL evaluation using the NIL of C9285

**Figure 7.**
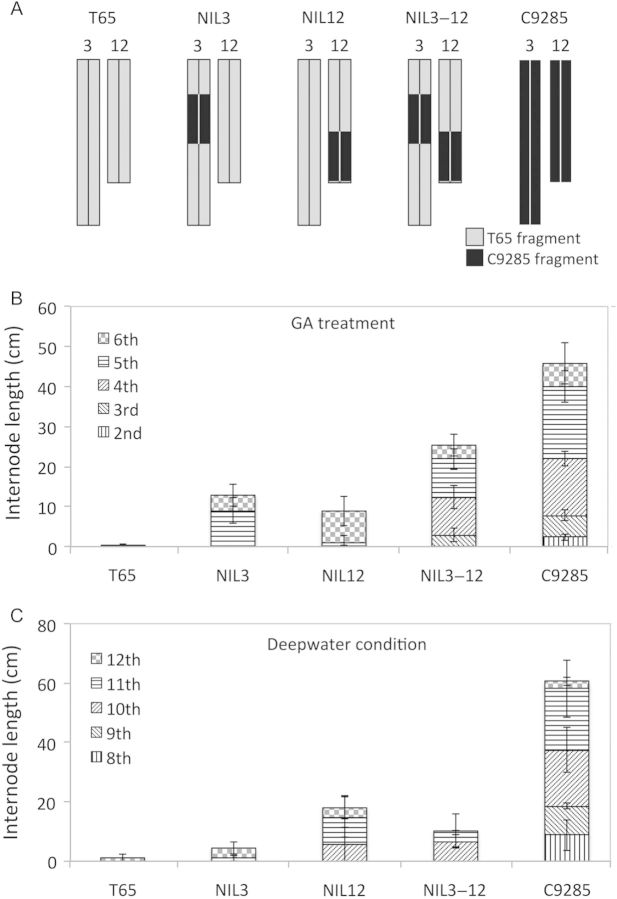
Evaluation of the QTLs on chromosomes 3 and 12. (A) Graphical genotypes of chromosomes 3 and 12. Grey and black boxes indicate T65 chromosomes and C9285 chromosomes, respectively. (B) Length of the elongated internode in response to GA. (C) Length of the elongated internode under deepwater conditions. Plants were submerged for 2 weeks and the internode lengths were measured. Error bars indicate ± SD (*n* = 6).

In our previous studies, we performed a QTL analysis of the deepwater response and detected NIL3, NIL12 and NIL3–12 carrying QTLs in another deepwater rice, C9285, in the T65 genetic background (Hattori *et al.* 2007, 2009). The QTL regions on chromosomes 3 and 12 in this study were partially consistent with those for the deepwater response in a previous study (Fig. 3; Hattori *et al.* 2007). Hence, we investigated the GA responsiveness of NIL3, NIL12 and NIL3–12 to confirm whether QTLs mediating the GA response are conserved in other deepwater rice. We treated the plants with GA and assessed the degree of internode elongation. T65 showed only slight internode elongation (0.3 cm) at the sixth internode (Fig. 7B). Because the internode length was <0.5 mm, the LEI and NEI of T65 were recorded as not detected. Meanwhile, NIL3, NIL12 and NIL3–12 induced internode elongation under GA treatment (Fig. 7B). NIL3 and NIL12 exhibited significant internode elongation under GA treatment (TIL = 12.9 and 8.7 cm, respectively). The LEI and NEI were 5 and 2 in both lines, respectively. NIL3–12 showed greater internode elongation (TIL, 25.3 cm; LEI, 3; NEI, 4) than NIL3 and NIL12. Under deepwater conditions, these NILs induced internode elongation (Fig. 7C). Therefore, the QTLs on chromosomes 3 and 12 may be common among deepwater rice, and the QTL on chromosome 3 of deepwater rice may have a stronger positive effect than the QTL on chromosome 12 on the response to GA.

## Discussion

Previously, ethylene-induced internode elongation in deepwater rice was shown to be caused by increased activity of endogenous GA (Raskin and Kende 1984). Additively, a genetic analysis using F_1_ plants of the GA biosynthesis-deficient mutant Tan-ginbozu (a weak allele of ent-kaurene oxidase) and Aswina (deepwater rice) suggested that GA is involved in internode elongation in deepwater rice because the F_1_ plants showed a semi-dominant internode elongation phenotype under deepwater conditions (Suge 1987). Kutschera and Kende (1988) also showed that GA controls the internode profile (e.g. extensibility and osmotic potential for internode elongation) of deepwater rice. Furthermore, the endogenous level of GA was increased in deepwater rice under deepwater conditions, but it did not occur in non-deepwater rice (Hattori *et al.* 2009). Non-deepwater rice induced internode elongation during heading time, and the expression of genes involved in GA biosynthesis and signalling factors has been observed in the elongated internodes of non-deepwater rice (Kaneko *et al.* 2003). Although previous studies have reported that GA is a critical component of internode elongation in rice, few reports have compared the GA responsiveness of non-deepwater and deepwater rice seedlings (Inouye and Kim 1985). First, to examine the GA responsiveness of non-deepwater rice and deepwater rice, we applied GA to non-deepwater rice and deepwater rice and measured the length of the second leaf sheath and internode. The second leaf sheath elongated in response to GA in both groups of plants (Fig. 1). Unexpectedly, whereas deepwater rice induced internode elongation in response to GA, non-deepwater rice did not (Fig. 1C). These results indicate the presence of factors that regulate internode-specific elongation in response to GA in deepwater rice seedlings. Given not only the deficiency in GA accumulation but also the absence of GA responsiveness in the internodes of non-deepwater rice seedlings, it appears that non-deepwater rice does not exhibit internode elongation in response to deepwater conditions and GA. Taking our results together, we assumed that a novel factor for GA responsiveness must exist.

In this study, CIM and a two-QTL scan for TIL, LEI and NEI detected significant QTLs (Figs 3 and 4, and Tables 1 and 2). Interestingly, QTLs were detected on chromosomes 3, 9 and 12 across three phenotypes by CIM and a two-QTL scan. To evaluate the effects of QTLs on chromosomes 3 and 12, we subjected NIL3, NIL12 and NIL3–12 to GA treatment (Fig. 7). Although plants carrying the QTL regions on chromosomes 3 and/or 12 were derived from the deepwater rice C9285, internode elongation was stimulated by GA treatment. In particular, NIL3–12, which has the QTLs on chromosomes 3 and 12, strongly induced internode elongation, as compared with NIL3 and NIL12, which have these QTLs singly. These results are consistent with the phenotypic effects in the T65/Bhadua RIL (Fig. 6). Since the NILs and RILs were derived from two different deepwater rice varieties, we conclude that these QTLs can lead to internode elongation in response to GA. In previous studies, QTLs of LEI related to deepwater response were detected on chromosomes 3 and 12 (Nemoto *et al.* 2004; Tang *et al.* 2005; Hattori *et al.* 2007; Kawano *et al.* 2008). Because LEI indicated correlations between TIL and NEI, it was proposed that LEI is the most important trait related to internode elongation ability under deepwater conditions (Inouye 1983). In addition, the number of non-elongated internodes was reduced by GA treatment (Takahashi 1991). This result implies that elongation of the lower internode was induced by GA. Additionally, the GA content of deepwater rice was elevated under deepwater conditions (Hattori *et al.* 2009). Although internode elongation in NIL3, NIL12 and NIL3–12 was induced under deepwater conditions, the magnitudes of the effects were slight when compared with GA treatment (Fig. 7). Taken together, our results suggest that chromosome regions in deepwater rice other than the QTL regions on chromosomes 3 and 12 participate in the accumulation of GA under deepwater conditions; the increase in GA responsiveness caused by the QTLs on chromosomes 3 and 12 promotes the onset of lower internode elongation. Consequently, TIL and NEI are enhanced in deepwater rice. It seems that the slight internode elongation of the NILs under deepwater conditions was due to the poor GA biosynthesis ability of these plants; in comparison, the *SK* genes in the QTL region on chromosome 12 caused the elongation of NIL12 and NIL3–12. To test this hypothesis, it is necessary to measure the GA content in the NILs under deepwater conditions. Meanwhile, *qGTIL4*, *qGLEI1*, *qGLEI12* and *qGNEI12* could not be detected without the QTL on chromosome 3 (Table 2). This result suggests that the QTL on chromosome 3 acts as a central regulator of GA responsiveness. Previously, *O. sativa*-*GROWTH-REGULATING FACTOR1* (*OsGRF1*) and 11 homologues were identified (van der Knaap *et al.* 2000; Choi *et al.* 2004). These genes were expected as transcription activators and their expressions were induced in response to exogenous GA and submergence. *OsGRF1* was expressed in the intercalary meristem of the internode in GA-treated or submerged rice, which preceded the expression of marker genes of cell division such as histone H3 and cyclin transcript *cycOs1* (van der Knaap *et al.* 2000). These results imply that *OsGRF1* is involved in GA-dependent stem elongation and meristem maintenance. Although *OsGRF6* and *OsGRF9* were found on chromosome 3, the positions were far from the QTL region on chromosome 3. Hence, the QTL on chromosome 3 is independent of *OsGRF*s. A notable QTL was not found in the QTL region on chromosome 9 in the Q-TARO database (http://qtaro.abr.affrc.go.jp/); thus, the QTL on chromosome 9 may be a new QTL that regulates internode elongation in response to GA. However, the sample size in this study was small to perform high-precision QTL analysis (*n* = 84). A small sample size can lead to the identification of false-positive QTLs. Except for the QTLs on chromosomes 3 and 12, further investigation is necessary to confirm the effects of QTLs on chromosome 9 as well as on the other chromosomes. We previously identified two genes located in the QTL on chromosome 12 that influence the deepwater response, *SK1* and *SK2*. Notably, *SK*s were absent in non-deepwater rice (Hattori *et al.* 2009). These *SK*s encode transcription factors possessing an AP2/ERF domain. Only ethylene treatment induced *SK* upregulation; exogenous GA and other phytohormones did not influence *SK* expression (Hattori *et al.* 2009). Although the position of the QTL on chromosome 12 in this study overlapped with the *SK* region, the peak of this QTL was at a distance (Fig. 3; Hattori *et al.* 2007, 2009). On the other hand, *OsGRF7* was located close to the peak. Hence, it is possible that *OsGRF7* on chromosome 12 regulates GA responsiveness for internode elongation.

Previous studies elucidated the GA signalling pathway and downstream factors. Among these, DELLA proteins, which are key regulators of GA signalling, have been characterized in genetic and biochemical studies (Ikeda *et al.* 2001; Itoh *et al.* 2002; Sasaki *et al.* 2003; Ueguchi-Tanaka *et al.* 2007). In the absence of GA, DELLA proteins function as repressors, leading to plant growth suppression. The DELLA proteins are degraded by 26S proteasome in the presence of GA, and plant growth is released from DELLA suppression. Further, recent reports have suggested that not only DELLA degradation but also DELLA interactions with other factors regulate plant growth, including hypocotyl elongation, GA-jasmonate or -ethylene crosstalk, and seed germination (Zhang *et al.* 2011; Cheminant *et al.* 2011; An *et al.* 2012; Yang *et al.* 2012). A DELLA-independent GA response has also been demonstrated (Fuentes *et al.* 2012). Based on these reports, the QTL on chromosome 3 and other QTLs may regulate the DELLA function or act downstream of GA signalling. It is also possible that these QTLs induce the formation of a structure that enables internodes to respond to GA.

## Conclusions

In conclusion, our data showed the presence of factors that regulate internode-specific elongation in response to GA in deepwater rice seedlings. The QTL analysis demonstrated that QTLs on chromosomes 3 and 9 are the main regulators of internode elongation and several QTLs are involved in internode elongation in response to GA (e.g. the QTL on chromosome 12). We previously produced mapping populations for the QTLs on chromosomes 3 and 12. Ongoing studies using these populations will identify genes at each QTL that are responsible for internode elongation in rice. In addition, our data may contribute to the breeding of rice that is adapted for survival in long-term flood areas by raising GA responsiveness.

## Sources of Funding

This work was supported by Grants in Aid for Scientific Research (22119007 to M.A.) from the Ministry of Education, Culture, Sports and Science. This work was also supported by the Japan Science and Technology Agency-Japan International Cooperation Agency within the framework of the SATREPS to M.A.

## Contributions by the Authors

K.N., Y.K., T.K., R.B.A. and T.K. measured the phenotypic values. H.Y. and A.Y. developed RILs. T.N. executed genotyping by BeadXpress. K.N. and M.A. performed QTL analysis.

## Conflicts of Interest Statement

None declared.

## Supporting Information

The following Supporting Information is available in the online version of this article –**Table S1.** Sequences of probe for single-nucleotide polymorphism (SNP) array.
